# LINC00662 sponges miR-107 accelerating the invasiveness and proliferation of glioma cells

**DOI:** 10.7150/jca.46381

**Published:** 2020-07-29

**Authors:** Jinsong Wu, Xiaolong Guo, Dongxiao Xu, Hongri Zhang

**Affiliations:** Department of Neurosurgery, The First Affiliated Hospital, and College of Clinical Medicine of Henan University of Science and Technology, Luoyang 471003 Henan China.

**Keywords:** glioma, LINC00662, miR-107, HMGB1, ceRNA

## Abstract

Increasing evidence revealed that the aberrant expression of long non-coding RNAs (lncRNAs) has been implicated in tumorigenesis. However, the role and mechanisms of LINC00662 in glioma have not been elucidated. Here, we show that upregulation of LINC00662 expression in glioma is associated with advanced clinical features and poor prognosis. Our results from loss-of-function assays suggest that LINC00662 silencing suppresses the proliferative and invasive abilities of glioma cells. *In vivo,* glioma growth was inhibited by depletion of LINC00662 in nude mice. Mechanistically, LINC00662 directly interacts with miR-107. The High-mobility group box 1 protein (HMGB1) is a known target of miR-107. Moreover, rescue assays reveal that HMGB1 overexpression (or miR-107 inhibition) reverses the glioma growth inhibition caused by LINC00662 knockdown. In conclusion, our results indicate that LINC00662 acts as an oncogene in glioma by modulating the miR-107/HMGB1 axis, suggesting that LINC00662 could be a novel therapeutic target for glioma treatment.

## Introduction

Glioma is an intracranial malignancy of the glial cells that originates in the neuroectoderm and has a high mortality rate 1. Glioma treatment involves a combination of immunotherapy, sophisticated surgical resection, and advanced chemoradiation 2, 3. Despite the great advances attained in the treatment of gliomas, its 5-year overall survival rate remains low due to a high rate of invasiveness and recurrence 4, 5. Thus, it is imperative to explore the molecular pathogenesis of glioma to identify effective treatment targets.

Long non-coding RNAs (lncRNAs) are transcripts consisting of >200 nucleotides 6, 7. It is widely shown that dysregulation of lncRNAs contributes to the pathogenesis of various diseases, including cancers 8, 9. For example, high expression of MALAT1 has been associated with the progression of aggressive renal cell carcinoma 10. Also, the expression of HOXA11-AS is upregulated in gastric cancerous cells, where it promotes their proliferation and invasion via scaffolding PRC2, LSD1, and DNMT1 11. The lncRNA EPIC1 exerts an oncogenic effect through interaction with MYC 12. However, the biological roles of the lncRNA LINC00662 in glioma are still unclear.

Mechanistically, lncRNAs function as competitive endogenous RNAs (ceRNAs). ceRNAs are lncRNAs that modulate the expression of specific genes by sponging miRNA 13, 14. For instance, HOXA11‐AS regulates the expression of MMP16 by sponging miR‐146b‐5p in renal cancer 15, while HNF1A-AS1 promotes invasiveness and proliferation of lung cancer cells by sponging miR-17-5p 16. The lncRNA NEAT1 contributes to resistance towards paclitaxel in ovarian cancer by modulating miR-194/ZEB1 signaling 17.

Here, we examined the role of LINC00662 in glioma and found that it promotes glioma deterioration by regulating the LINC00662/miR-107/HMGB1 axis, suggesting its potential as a novel therapeutic target against glioma.

## Materials and Methods

### Patients and tissue samples

In summary, 41 glioma specimens and 11 normal brain specimens were collected from patients admitted to The First Affiliated Hospital, and College of Clinical Medicine of Henan University of Science and Technology. No patients received chemotherapy or radiotherapy before surgery. Informed written consent was obtained from all the patients before the study commenced. Isolated tissues were immediately frozen in liquid nitrogen immediately after sampling. Ethical clearance for this study was provided by the Medical Ethics Committee of our hospital (Approval no. 2019-A0023). Glioma patients' information was shown in Table 1.

### Cell culture and transfection

NHA (Normal human astrocytes) and six glioma cell lines (U87, A172, LN18, LN229, U118, and N251) were procured from the Chinese academy of sciences (Shanghai, China). These cells were grown in RPMI-1640 (Hyclone, USA) supplemented with 10% FBS (Gibco, USA) in a humidified incubator at 37ºC, 5% CO_2._

MiR-107 mimics, and miR-NC were designed by GenePharma (Shanghai, China), while siRNA against LINC00662 (si-LINC00662#1: 5′-GCAGGCGTACAACTAACAAdTdT-3′, si-LINC00662#2: 5′-TAAATTTTGTAATAAAATATAAGTAGA-3′, and si-LINC00662#3: 5′-GCUGCUGCCACUGUAAUAATT-3′) and negative control siRNA (si-NC) were bought from Ribobio. The above-mentioned oligonucleotides and plasmids were transfected to the cells using lipofectamine 3000 reagents (Invitrogen, USA).

### Quantitative real-time PCR

Cells were homogenized with TRIzol reagent (Invitrogen) to isolated total RNA and reverse transcribed using a commercial reverse transcription kit (Takara, Tokyo, Japan). RT-qPCR was performed on Bio-Rad CFX96 system (Bio-Rad, USA) using SYBER-Green Real-Time PCR Kit (Roche, Germany). Fold expression in mRNA levels were estimated with the 2^-ΔΔCt^ method. The PCR cycle parameters were as following: denaturation: 95°C, 30 s, 40 cycles; annealing: 60°C, 30 s; extension: 72°C, 30 s. Primer were as following: LINC00662, 5′-TGGACATCTGTCTGGAGG-3′ (forward) and 5′- GGCTGAGGCATAAGAATCG-3′ (reverse); miR-107, 5′-AGCAGCATTGTACAGGGCTATCA-3′ (forward) and 5′- GCGAGCACAGAATTAATACGAC-3′ (reverse); HMGB1, 5′-CCAGCGATAGTCCCACTGAT-3′ (forward) and 5′- CCTTCTCCTTGGCAGACATC-3′.

### CCK-8 assays

After transfection, cells were titrated onto 96-well plates, and 10 μL of CCK-8 solution (Dojindo, Japan) was added into each well. The optical density of each plate was read by a microplate reader (wavelength = 450 nm; Bio-Rad).

### Colony formation assay

Transfected glioma cells were cultured in 6-well plates for 14 days then mixed with 4% formaldehyde (Sigma). Next, cells were stained with 0.1% crystal violet (Sigma), after which the number of colonies formed was assessed manually.

### Wound healing assay

Migration was tested by wound healing assay. Transfected cells were plated in 12-well dishes reaching a confluence of 80%. Then cells were scratched across the surface of well by a pipette. After 24 h, the scratches were observed.

### Transwell invasion assay

Transfected glioma cells were plated in the upper chamber of Matrigel-coated membranes (BD, USA), and the lower chamber was filled with 10% FBS culture medium. After 24 h, transfected cells in the lower chamber were fixed (by methanol) and stained with crystal violet (0.1%).

### Subcellular fractionation

The part of nuclear and cytoplasm were extracted by Nuclear and Cytoplasmic RNA Purification Kit. Then, the relative expressions of LINC00662, GAPDH and U6 in cytoplasm or nuclear of the cells were explored by RT-qPCR assay. U6 and GAPDH were served as endogenous controls.

### Dual-luciferase assay

Plasmids harboring the predicted binding sites or mutant sites were purchased from Promega (Hangzhou, China), while plasmids containing the sequences for miR-107 were purchased from Sangon (Shanghai, China). MiR-107 mimics were co-transfected with LINC00662-Wt, LINC00662-Mut, HMGB1-Wt, or HMGB1-Mut using lipofectamine 3000. Dual-Luciferase Reporter Assay System (Promega) was used to explore the luciferase activity.

### RNA immunoprecipitation (RIP) assay

The RIP assay was done by a Magna RIP kit (Millipore) according to the manufacturer's instruction. The lysates were incubated with magnetic beads with anti-IgG as control and anti-Ago2 in RIP buffer. The levels of LINC00662 and miR-107 in the precipitates were quantified by RT-qPCR.

### RNA pull-down assay

Cell lysates of U87 and N251 cells were incubated with LINC00662 no-biotin probe or LINC00662 biotin probe and streptavidin beads (Invitrogen) were later added. RT-qPCR was applied to analyze expression levels of various miRNAs, separately.

### Western blot analysis

Western blot analysis was performed according to a previously described method 10.

### Xenograft mouse model

U87 cells (5 × 10^6^) were stably transfected with sh-LINC00662 or control vector (sh-NC) were subcutaneously injected to the back of female BALB/c nude mice (5-week-old, n=5, Nanjing biomedical research institute of Nanjing University). The size of tumors was monitored weekly by measuring the length (L) and width (W) using a caliper. Tumor volume (V) was calculated using the formula: V = 1/2×L×W^2^. After 6 weeks following injection, the mice were euthanized, and tumors were harvested and weighed. The protocol used for animal experiments was authorized by our hospital.

### Bioinformatics analysis

The levels of LINC00662 and HMGB1 in glioma was analyzed using GEPIA database. The Starbase 3.0, TargetScan and miRanda databases were applied to predict the target miRNAs of LINC00662. Besides, the target gene of miR-107 were predicted mircode, Targetscan, miRTarBase, and MicroT-CDS databases. The prediction outcomes were integrated by Venny2.1. The correlation between LINC00662 and miR-107 was predicted by Starbase 3.0 databases.

### Statistics analysis

All data were expressed as the mean ± standard deviation (SD) of 3 independent experiments. Data analysis was determined by using SPSS17.0. One-way ANOVA or student's t-test was used to assess for statistical significance between the treatment means of different treatments. *P* <0.05 was considered statistically significant.

## Results

### LINC00662 is upregulated in glioma

**Figure 1:**
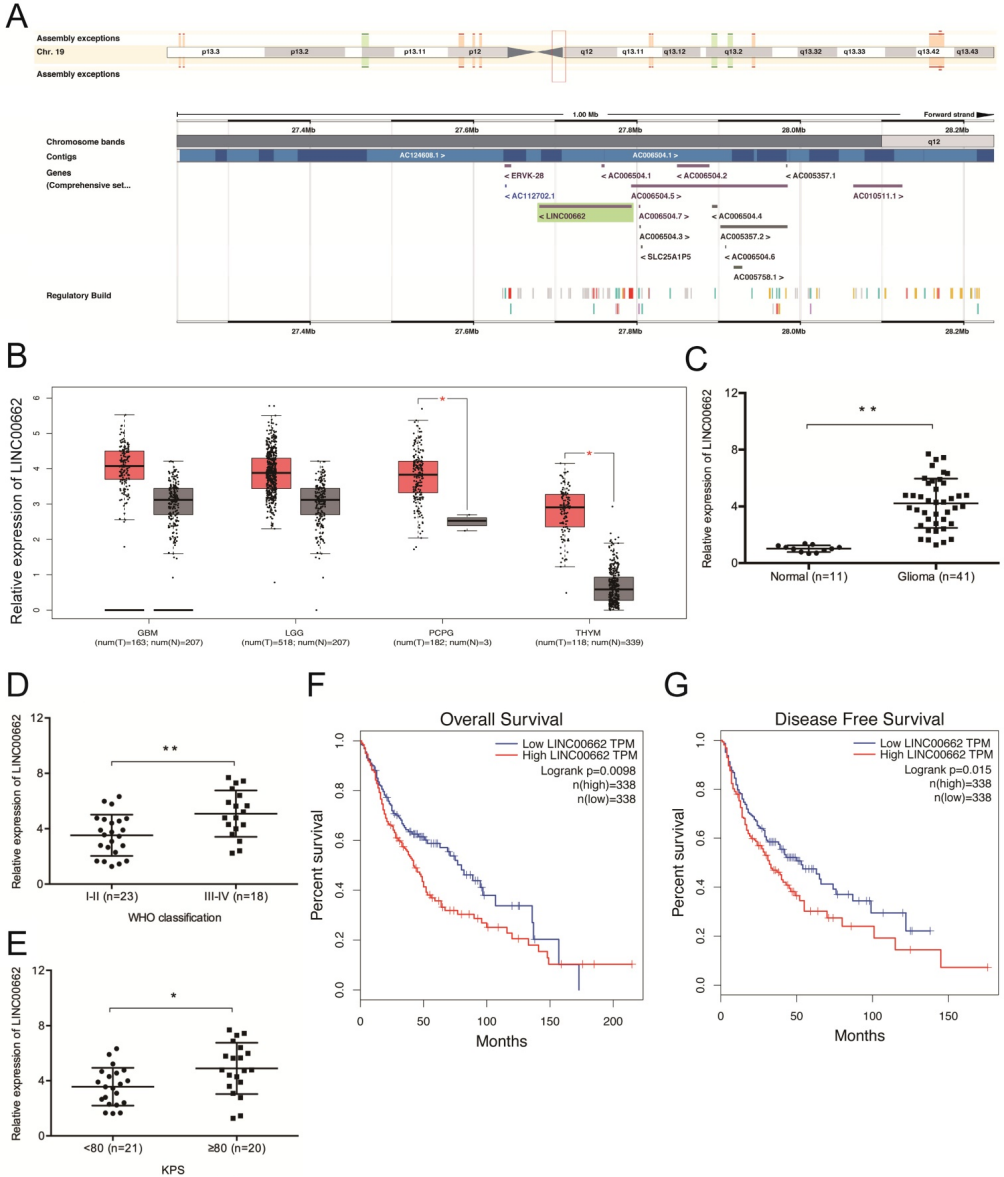
** LINC00662 expression is upregulated in glioma.** (**A**) The locus map of LINC00909 in the chromosome. (**B**) TCGA analysis of LINC00662 expression in glioma. (**C**) RT-qPCR analysis of LINC00662 expression in glioma specimens. (**D, E**) High LINC00662 expression correlates with advanced tumor grade and high KPS in patients. (**F, G**) High LINC00662 levels predict poor OS and DFS in glioma patients. GMB: Glioblastoma multiforme; LGG: Brain Lower Grade Glioma; PCPG: Pheochromocytoma and Paraganglioma; THYM: Thymoma. OS: overall survival; DFS: disease-free survival. *p <0.05, **p <0.01.

Sequence alignment by the NCBI BLAST revealed that LINC00662 is located on chromosome 19q12.3 and spans between nucleotides 27,678,814 to 27,796,263 (Figure 1A). We then explored LINC00662 expression in glioma by analyzing data from TCGA (The Cancer Genome Atlas) database and found that the expression of LINC00662 is dramatically elevated in glioma tissues (Figure 1B). The results were further confirmed in glioma patients (Figure 1C). High levels of LINC00662 are associated with advanced WHO grade and high Karnofsky performance score (KPS) (Figure 1D-E). GEPIA (Gene Expression Profiling Interactive Analysis) revealed that the OS (overall survival) and DFS (disease-free survival) rates of glioma patients with high LINC00662 expression are significantly lower (Figure 1F-G). These results indicate that the aberrant expression of LINC00662 is associated with the development and progression of glioma.

### LINC00662 knockdown reduces glioma cell proliferation and invasion

**Figure 2:**
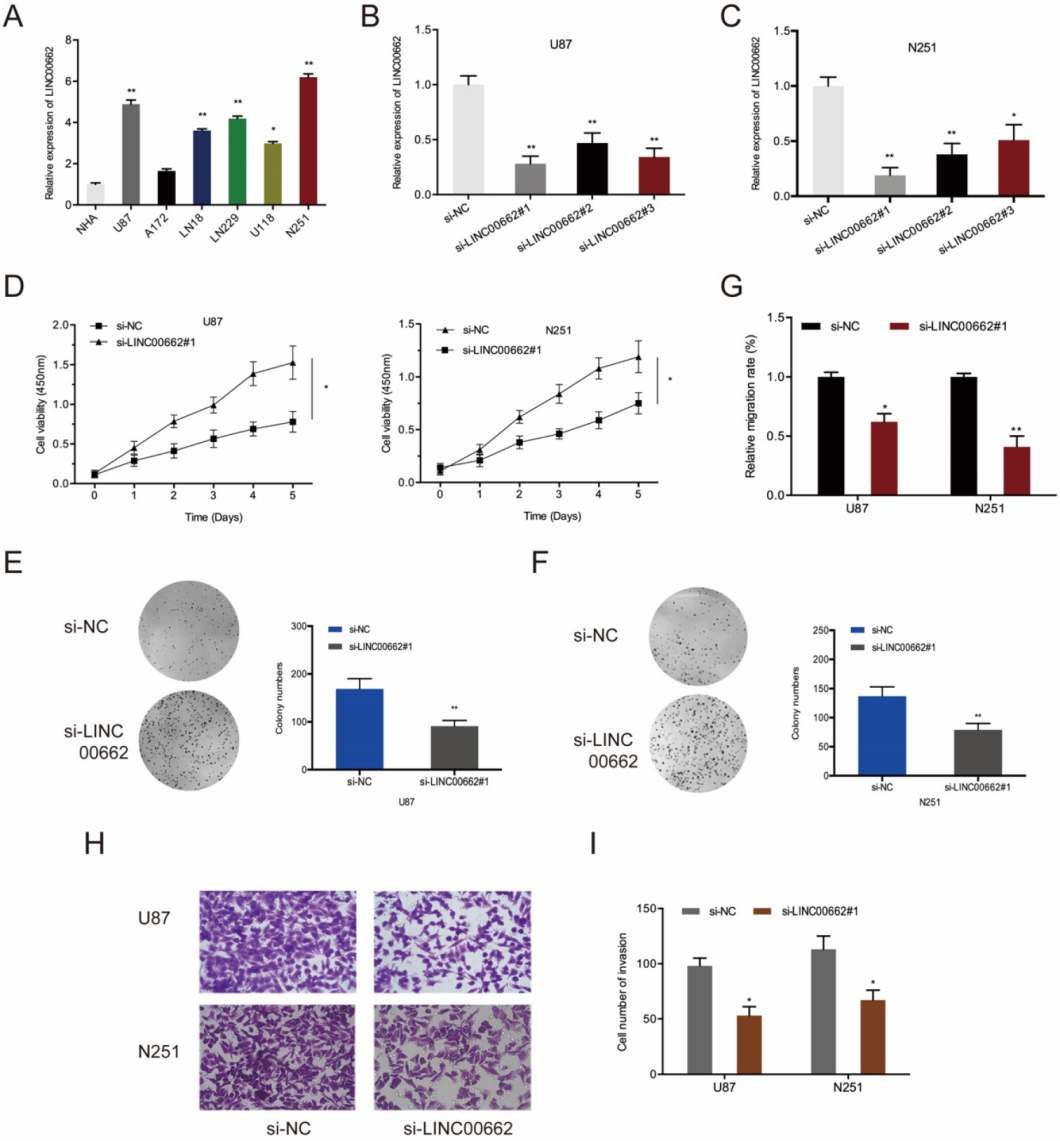
** LINC00662 suppression inhibits cell proliferation and invasion *in vitro*.** (**A**) LINC00662 expression in glioma cell lines. (**B, C**) LINC00662 expression in U87 and N251 cells transfected with si-LINC00662. (**D-F**) Cell viability by CCK8 and colony formation assays. (**G**) Assessment of cell migration by a wound-healing assay. (**H, I**) Cell invasion analysis by Transwell invasion assay. *p <0.05, **p <0.01.

Next, qRT-PCR showed that LINC00662 is markedly upregulated in glioma cells (U87, A172, LN18, LN229, U118, and N251) relative to normal human astrocytes (NHA) (Figure 2A). To determine if LINC00662 participates in the tumorigenesis of glioma, LINC00662 was deleted in U87 and N251 cells using si-LINC00662 (Figure 2B-C). The results of CCK8 and colony formation assays revealed that LINC00662 silencing significantly reduces the cell viability of U87 and N251 cells (Figure 2D-F). Besides, wound-healing and Transwell invasion assays suggested that knockdown of LINC00662 inhibits the migration and invasion of glioma cells (Figure 2G-I). These results suggested that LINC00662 could be oncogenic during glioma progression.

### LINC00662 sponges miR-107 in glioma

**Figure 3:**
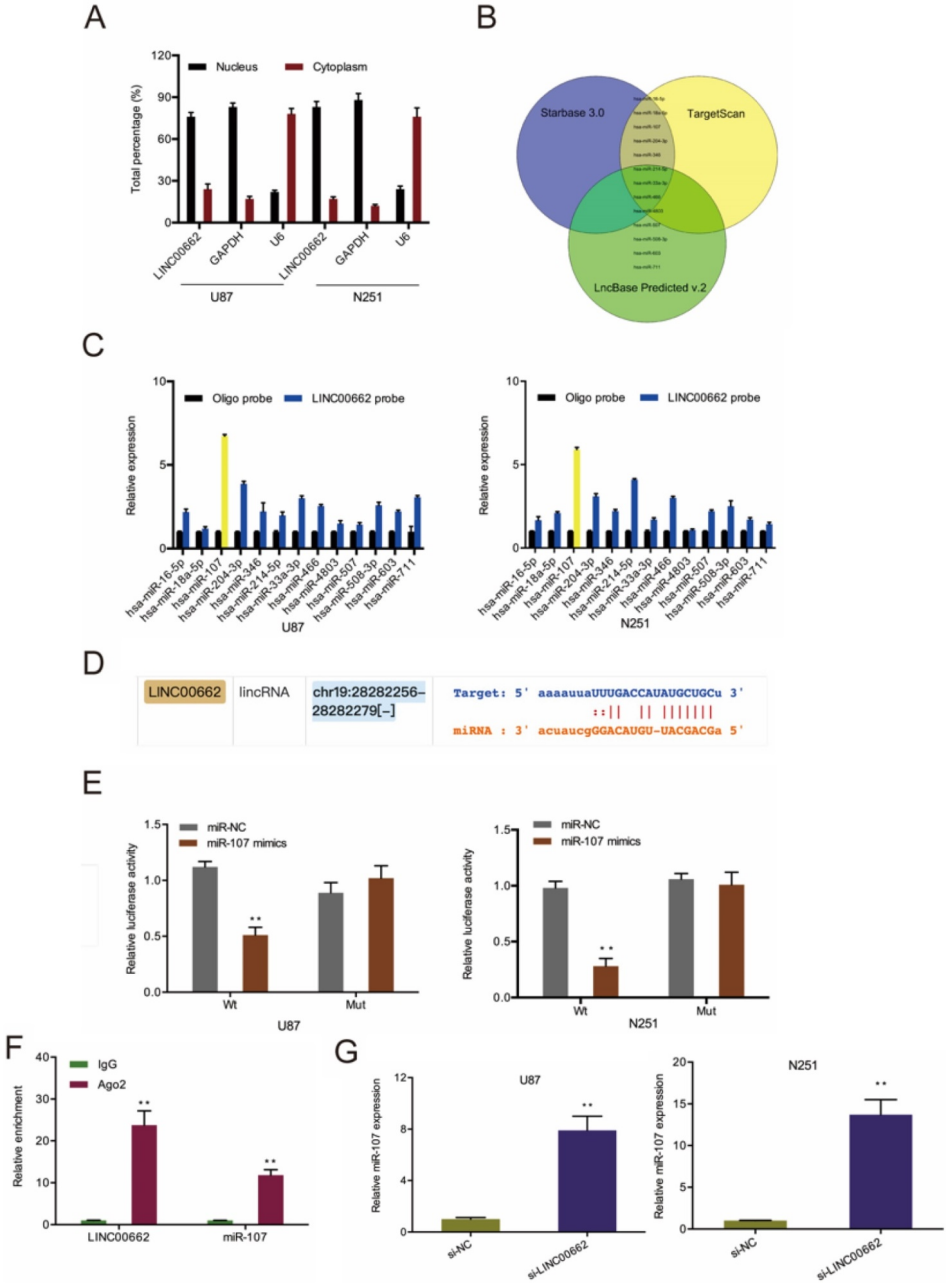
** LINC00662 directly binds to miR-107 in glioma.** (**A**) The cellular sub-localization of LINC00662 in U87 and N251 cells. (**B**) Bioinformatics explored the potential target of LINC00662. (**C, D**) RNA pull-down assay confirmed that LINC00662 could bind to miR-107. (**E, F**) The interaction between LINC00662 and miR-107 was confirmed by luciferase reporter analysis and RIP assay. (**G**) LINC00662 depletion enhanced miR-107 expression in U87 and N251 cells. *p<0.05, **p <0.01.

To understand the underlying mechanism of LINC00662, we conducted a cell fractionation assay to evaluate its subcellular localization. Results revealed that LINC00662 is mainly located in the cytoplasm (Figure 3A), suggesting that LINC00662 might act as a miRNA sponge in glioma. Next, we used Starbase 3.0, TargetScan, and miRanda to explore the potential targets of LINC00662 (Figure 3B) and uncovered that miR-107 was the most enriched in RNA pull-down assays (Figure 3C-D). Luciferase reporter assay further confirmed the interaction between LINC00662 and miR-107 (Figure 3E). RIP analysis revealed LINC00662, and miR-107 co-immunoprecipitate in Ago2 complex (Figure 3F), and RT-qPCR analysis revealed that depletion of LINC00662 enhances miR-107 levels in glioma cells (Figure 3G).

**Figure 4:**
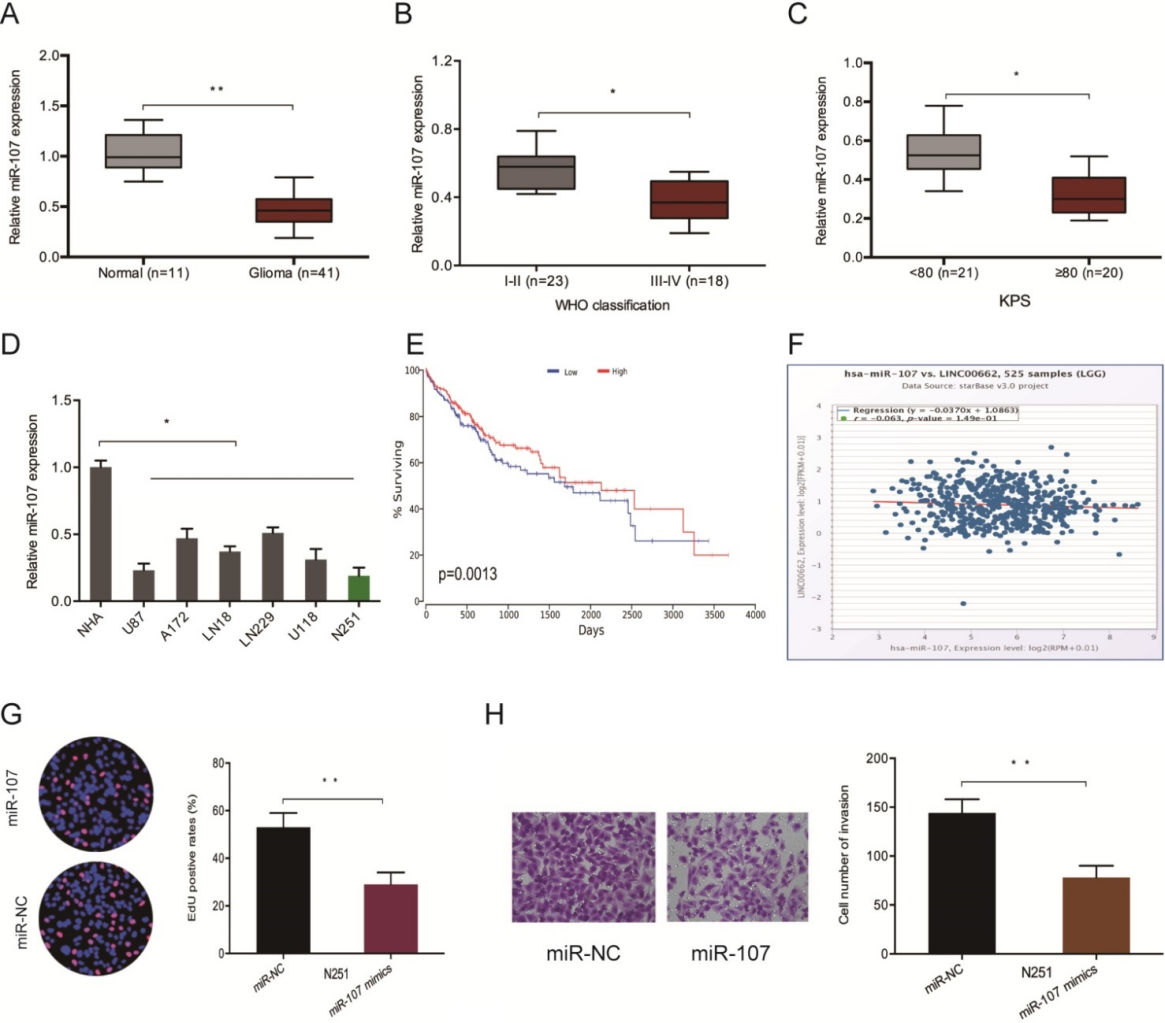
** LINC00662 promotes glioma deterioration by sponging miR-107.** (**A**) miR-107 levels in glioma specimens as evaluated by RT-qPCR. (**B, C**) Low miR-107 expression correlates with advanced WHO grade and high KPS score in patients. (**D**) miR-107 expression negatively correlates with LINC00662 expression in glioma patients. (**E, F**) Low miR-107 levels negatively correlated with poor OS and high LINC00662 expression in glioma patients. (**G, H**) Edu and invasion assays showed that miR-107 overexpression reduced glioma cells proliferation and invasion. *p <0.05, **p <0.01.

Furthermore, we evaluated the levels of miR-107 in glioma samples to confirm whether LINC00662 regulates glioma progression by interacting with miR-107. The RT-qPCR analysis demonstrated that the expression of miR-107 is repressed in glioma tissues and cell lines (Figure 4A and 4D). Low miR-107 levels negatively correlated with advanced WHO grade (Figure 4B), high KPS score (Figure 4C), poor OS (Figure 4E) and high LINC00662 expression (Figure 4F) in patients. Moreover, function experiments reported that miR-107 mimics reversed N251 cells proliferation and invasive abilities *in vitro* (Figure 4G-H). These observations indicated that LINC00662 sponges miR-107 in glioma.

### HMGB1 is a target of miR-107

**Figure 5:**
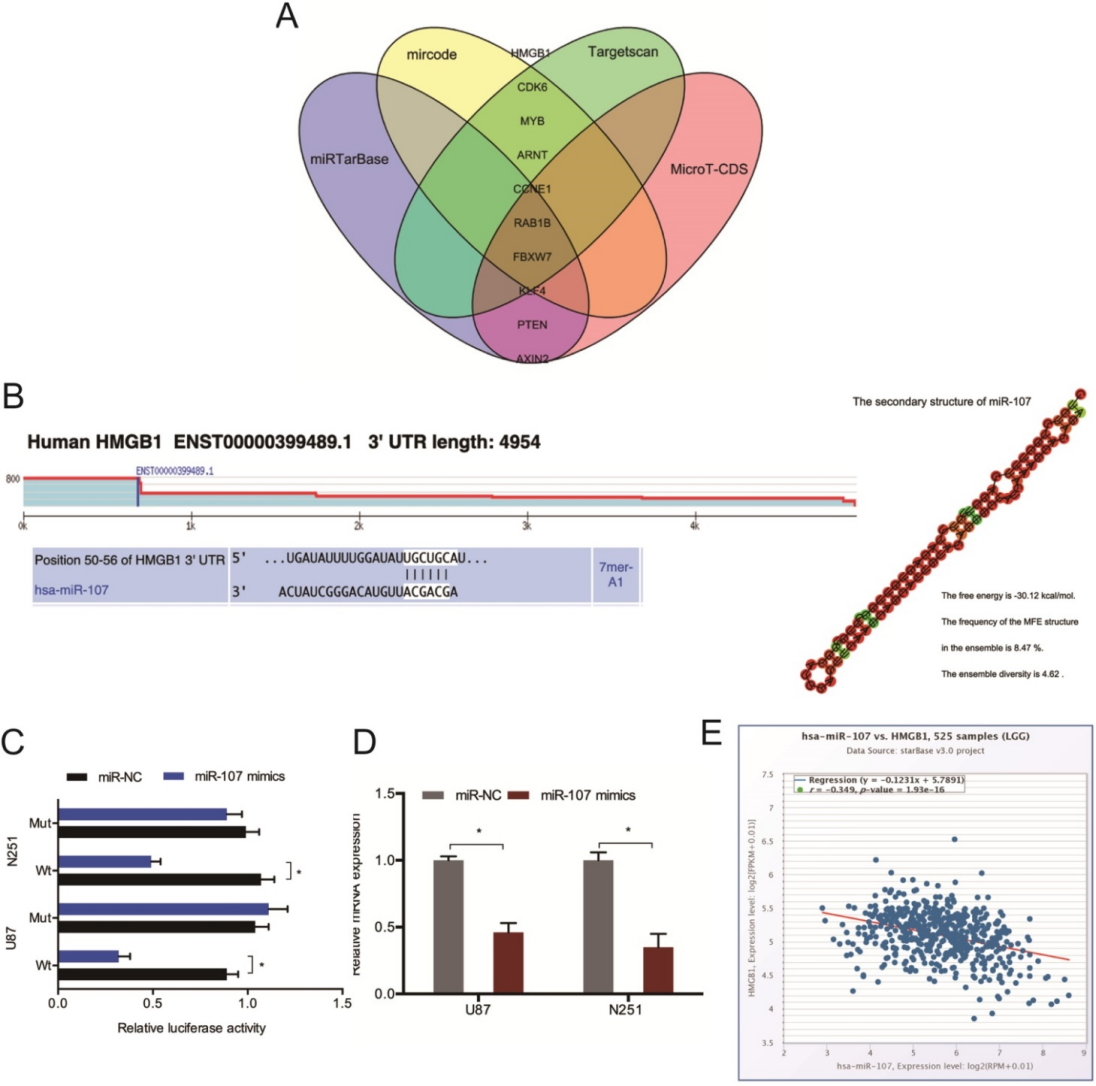
** HMGB1 acts as a target of miR-107.** (**A**) Bioinformatic analysis predicted the latent binding sites of miR-107. (**B**) Schematic diagram of the interacting sites. (**C**) The interaction between miR-107 and HMGB1 was confirmed by luciferase reporter analysis. (**D**) miR-107 mimics reduced HMGB1 expression in glioma cells. (**E**) HMGB1 expression was negatively correlated with miR-107 expression in patients. *p<0.05, **p <0.01.

Next, we conducted mircode, Targetscan, miRTarBase, and MicroT-CDS analysis to identify the target of miR-107, and HMGB1 was chosen as a target of miR-107 (Figure 5A). The binding sites for HMGB1 bind to miR-107 were shown in Figure 5B. And the luciferase reporter assay was used to experimentally validate the interaction between HMGB1 and miR-107 (Figure 5C). The RT-qPCR analysis revealed that miR-107 mimics decreased the expression of HMGB1 in glioma cells (Figure 5D). Moreover, low miR-107 levels negatively correlated with high HMGB1 expression in glioma (Figure 5E).

**Figure 6:**
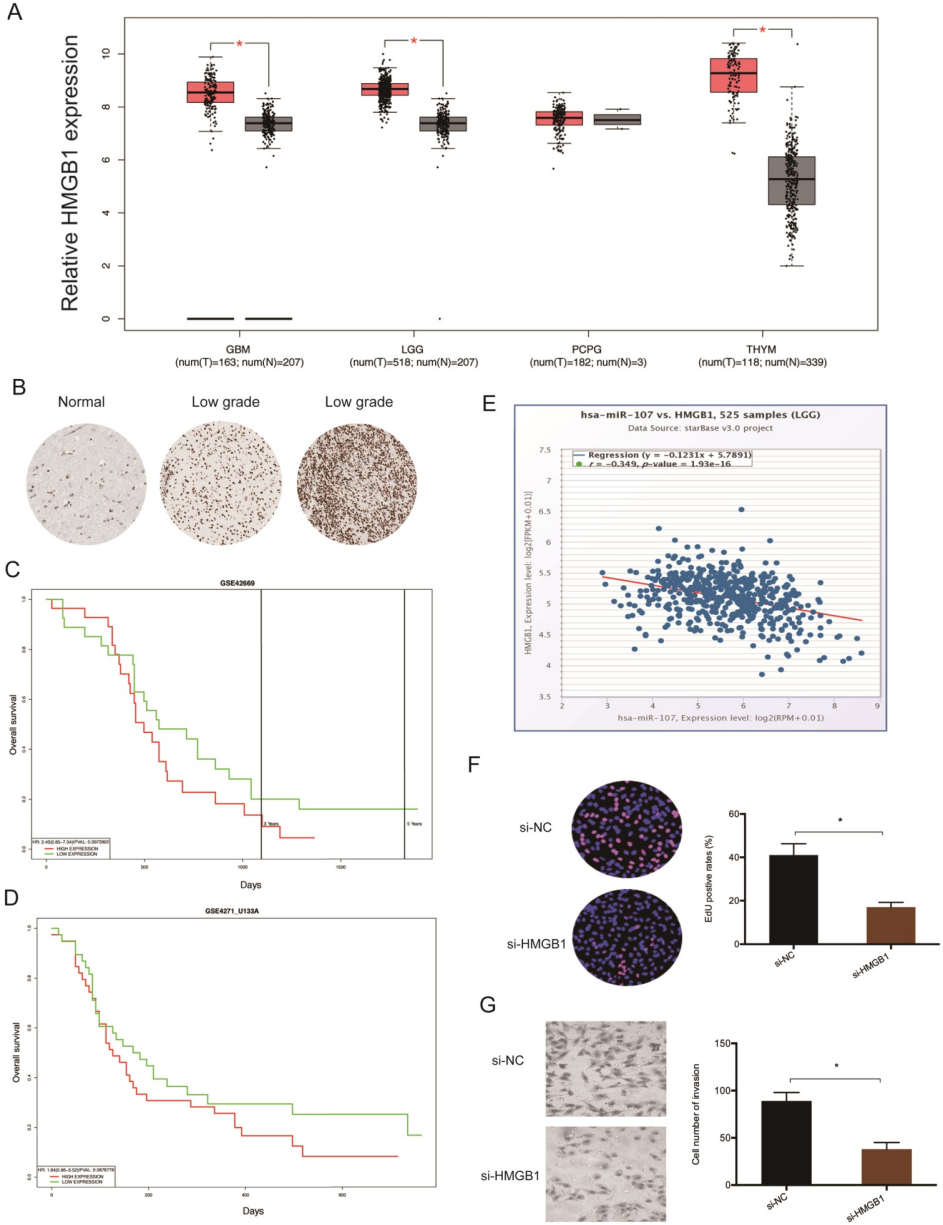
** HMGB1 is upregulated in glioma.** (**A**) Analysis of TCGA data for HMGB1 expression in glioma tissues. (**B**) Evaluation of HMGB1 expression in glioma tissues by IHC. (**C, D**) High HMGB1 expression correlates with poor prognosis. (**E**) HMGB1 expression negatively correlates with miR-107 expression in patients. (**F, G**) HMGB1 suppression reduces glioma cells proliferation and invasion *in vitro*. *p<0.05, **p <0.01.

Analysis of data extracted from TCGA revealed that glioma tissues have elevated levels of HMGB1 (Figure 6A). This finding was validated by immunohistochemistry (IHC) (Figure 6B). Kaplan-Meier tests revealed that high HMGB1 levels correlate with poor glioma prognosis (Figure 6C-D), and correlation analysis showed that HMGB1 levels inversely correlate with miR-107 expression in glioma tissues (Figure 6E). Moreover, functional tests revealed that HMGB1 suppression represses the proliferation invasion of glioma cells *in vitro* (Figure 6F-G). Taken together, these data indicated that HMGB1 could be a target of miR-107 in glioma.

### LINC00662 regulates glioma progression via the miR-107/HMGB1 axis

**Figure 7:**
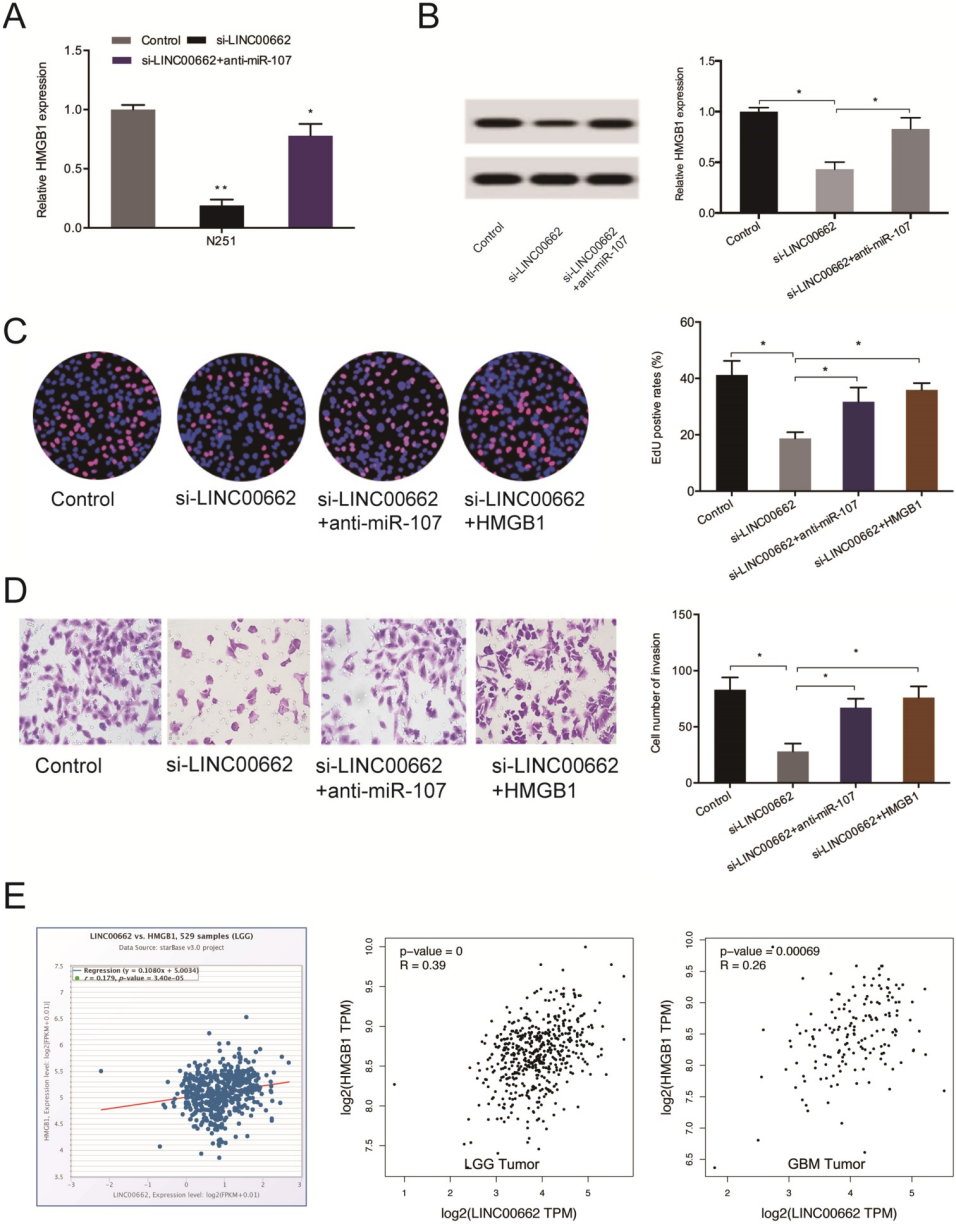
** LINC00662 regulates glioma progression via the miR-107/HMGB1 axis.** (**A, B**) miR-107 inhibitors abolish the effects of LINC00662 suppression on HMGB1 expression at the mRNA and protein levels. (**C, D**) HMGB1 overexpression reverses the effect of LINC00662 suppression on glioma cells proliferation and invasion. (**E**) HMGB1 expression positively correlates with LINC00662 expression in patients. *p < 0.05, **p <0.01.

Next, we observed that the silencing of LINC00662 decreases the levels of HMGB1 both at the mRNA and protein levels and the effects could be abolished by miR-107 inhibition (Figure 7A-B). 5-Ethynyl-2´-deoxyuridine (EdU) assay revealed suppressed cell proliferation upon silencing of LINC00662, which could be countervailed by HMGB1 overexpression (or miR-107 inhibitors) (Figure 7C). Cell invasiveness was suppressed by si-LINC00662, and the suppression was reversed by HMGB1 overexpression (or miR-107 inhibitors) (Figure 7D). Moreover, correlation analysis revealed that HMGB1 positively correlates with the expression of LINC00662 in glioma tissues (Figure 7E).

### LINC00662 increases tumor growth *in vivo*

**Figure 8:**
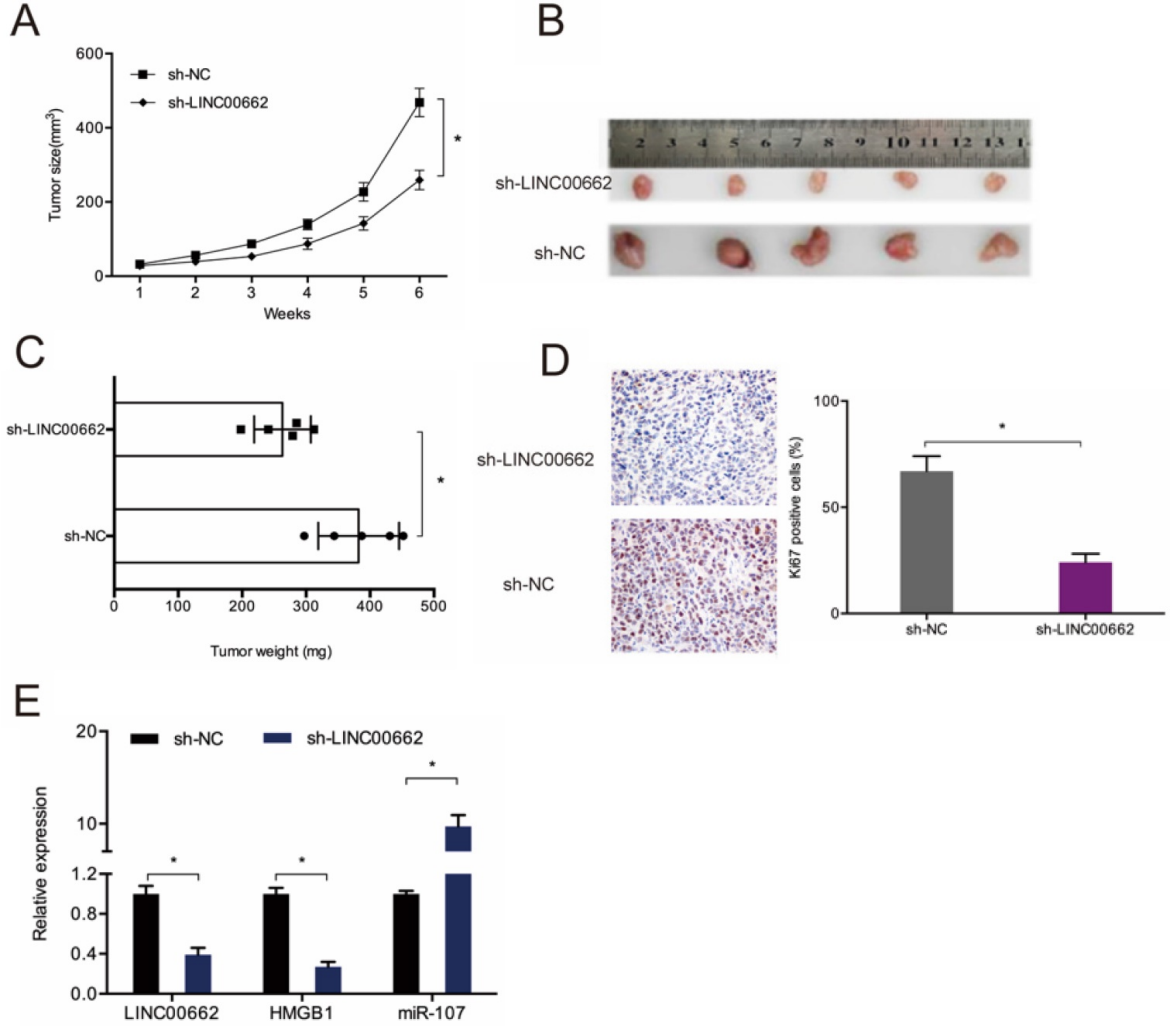
** LINC00662 knockdown reduces tumor growth *in vivo*.** (**A**) Tumor growth curve in mouse glioma cell xenografts. (**B, C**) Analyses of tumor volume and weight. (**D**) LINC00662 suppression reduces Ki67 expression in mice tissues. (**E**) RT-qPCR analysis of tumor miR-107 and HMGB1 expression. *p<0.05, **p <0.01.

**Figure 9:**
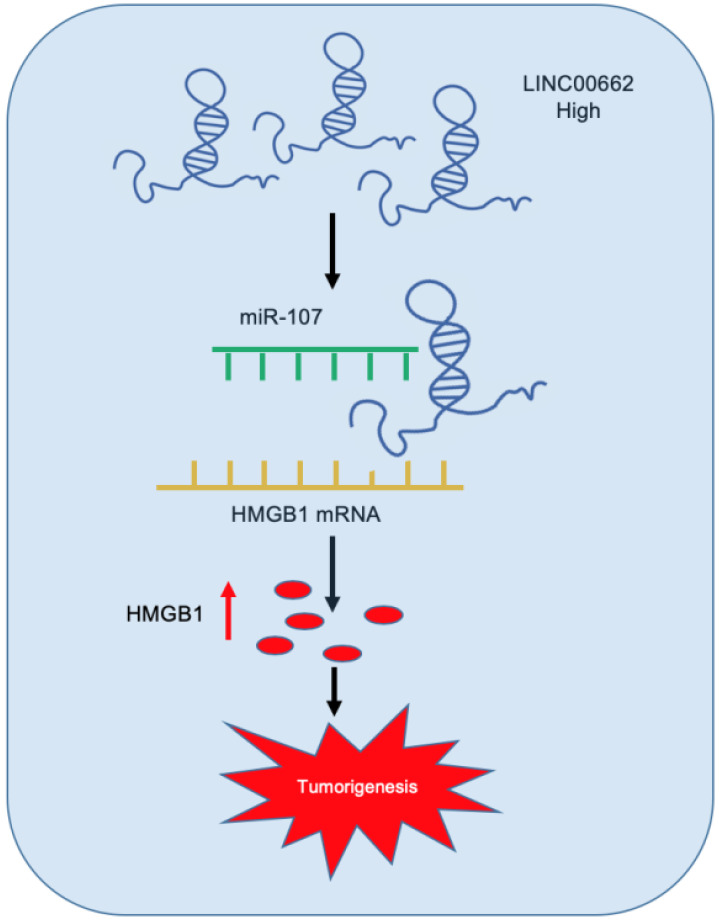
Schematic representation of LINC00662/miR-107/HMGB1 axis in glioma.

To assess the role of LINC00662 in tumor development *in vivo*, sh-LINC00662 was transfected into U87 cells and xenografted into nude mice, and we found that LINC00662 suppression slows tumor growth *in vivo* (Figure 8A-B). Tumor weight assessment revealed that LINC00662 knockdown tumors are much smaller than control group (Figure 8C). IHC analysis results showed that LINC00662 suppresses proliferation, as revealed by reduced Ki67 staining in xenograft tumors (Figure 8D). RT-qPCR analysis revealed that miR-107 is upregulated in LINC00662 knockdown mice, while the expression of HMGB1 is reduced (Figure 8E). Thus, LINC00662 could accelerate glioma deterioration by modulating the miR-107/HMGB1 axis (Figure 9).

## Discussion

Long non-coding RNAs (lncRNAs) have been implicated in glioma tumorigenesis. For instance, the upregulation of DANCR promotes glioma progression via Wnt signaling 18, and SNHG3 promotes glioma development by suppressing the expression of KLF2 and p21 expression 19. Meanwhile, CASC2 couples with miR‐181a, thereby modulating resistance to Temozolomide (TMZ) and glioma growth via PTEN signaling 20. Although the correlation between multiple lncRNAs and glioma has been reported previously, their functions and regulatory mechanisms are largely obscure. Therefore, the present study uncovered the potential role and molecular mechanism of the glioma-related lncRNA, LINC00662.

Recently, multiple studies have implicated LINC00662 was dysregulated and had potential clinical significance in tumors. For example, Xu et al. showed that LINC00662 promoted oral squamous cell carcinoma cells proliferation and migration abilities 21. Liu et al. showed that high LINC00662 expression contributed acute myeloid leukemia cells growth via targeting the miR-340-5p/ROCK1 axis 22. Wang et al. showed that LINC00662/miR-497-5p/AVL9 axis promoted colorectal cancer tumorigenesis 23. Consistent with earlier studies, we showed LINC00662 expression is significantly overexpressed in clinically advanced gliomas. Additionally, inhibition of LINC00662 suppresses the proliferation abilities and invasion abilities of glioma cells.

As the lncRNA-miRNA-mRNA axis is the most common mechanism involved in the regulation of lncRNAs, we sought to identify the possible miR-107 target by bioinformatics analysis. Recently, Takahashi et al. showed that miR-107 induced cell cycle arrest in lung cancer cell lines 24. Chen et al. showed that miR-107 suppressed glioma progression by modulating Notch2 expression 25. Moreover, Zheng et al. showed that NEAT1 suppression inhibited glioma progression via regulating the miR-107/CDK14 axis 26. However, the effects and mechanisms of miR-107 on glioma progression is obscure. Here, we show that LINC00662 directly interacts with miR-107 in the Ago2 complex and presented negative correlation in glioma tissues. Additionally, miR-107 knockdown partially reversed the inhibition of glioma cell proliferation and invasion caused by LINC00662 silencing. These finding indicates that miR-107 might modulate glioma development by acting as a ceRNA.

The high mobility group box 1 (HMGB1) is a highly conserved DNA-binding protein that translocates to the nucleus to interact with histone nucleosomes and transcriptional factors 27. Multiple studies have shown that HMGB1 drives cancer development, including glioma 28-30. The present study has shown that the upregulation of HMGB1 in glioma tissues is correlated to poor prognosis. Additionally, we found that LINC00662 upregulates HMGB1 to exert its oncogenic role. Taken together, these data highlight the potential of targeting LINC00662 therapeutically against glioma.

In conclusion, our study uncovered an oncogenic role and mechanism of LINC00662 in the development and progression of glioma. LINC00662 might exert its functions by regulating the miR-107/HMGB1 axis in glioma. Therefore, our data highlights the potential of this lncRNA as an anti-glioma therapeutic target.

## Highlights

- LINC00662 was upregulated in glioma;
- LINC00662 promoted glioma cell proliferation and invasion;
- LINC00662 enhanced glioma progression by regulating the miR-107/HMGB1 axis.

## Authors' Contributions

JSW, HRZ designed the research; JSW, XLG, DXX performed the experiments and analyzed the data; JSW, HRZ wrote the manuscript. All authors read and approved the final manuscript.

## Figures and Tables

**Table 1:** Clinicopathological characteristics of glioma patients

Clinical features	Group	Total
Gender	Male	22
	Female	19
Age (years)	<50	16
	≥50	25
WHO grade	I/II	23
	III/IV	18
KPS	<80	21
	≥80	20

## Abbreviations

LncRNAs: Long non-coding RNAs
miRNAs: MicroRNAs
HMGB1: High Mobility Group Box 1
ceRNA: Competing endogenous RNA
AGO2: Argonaute 2
3'UTR: 3′-untranslated region
ANOVA: Analysis of variance
RIP: RNA immunoprecipitation
CCK-8: Cell Counting Kit-8
GAPDH: Glyceraldehyde phosphate dehydrogenase
RIPA: Radio-immunoprecipitation assay
SDS-PAGE: sodium dodecyl sulphate-polyacrylamide gel electrophoresis
OD: Optical density
TCGA: The Cancer Genome Atlas
DFS: disease-free survival
OS: overall survival
